# Nitric Oxide and Small and Intermediate Calcium-Activated Potassium Channels Mediate the Vasodilation Induced by Apigenin in the Resistance Vessels of Hypertensive Rats

**DOI:** 10.3390/molecules29225425

**Published:** 2024-11-18

**Authors:** Lislaine Maria Klider, Maria Luiza Fidelis da Silva, Gustavo Ratti da Silva, João Ricardo Cray da Costa, Marcia Alessandra Arantes Marques, Emerson Luiz Botelho Lourenço, Francislaine Aparecida dos Reis Lívero, Jane Manfron, Arquimedes Gasparotto Junior

**Affiliations:** 1 Laboratory of Cardiometabolic Pharmacology, Postgraduate Program in Pharmacology (UFPR), Federal University of Paraná, Curitiba 81531-980, PR, Brazil; lis_klider19@hotmail.com (L.M.K.);; 2 Laboratory of Cardiovascular Pharmacology (LaFaC), Faculty of Health Sciences, Federal University of Grande Dourados (UFGD), Dourados 79804-970, MS, Brazil; m.alufidelis@hotmail.com; 3 Laboratory of Preclinical Research of Natural Products, Post Graduate Program in Animal Science with Emphasis on Bioactive Products, Paranaense University, Umuarama 87502-210, PR, Brazil; gustavo_ratti@hotmail.com (G.R.d.S.); doutoramarciamarques@gmail.com (M.A.A.M.); emerson@prof.unipar.br (E.L.B.L.); 4 Laboratory of Preclinical Research of Natural Products, Post-Graduate Program in Medicinal Plants and Phytotherapeutics in Basic Attention, Paranaense University, Umuarama 87502-210, PR, Brazil; joao.cray@edu.unipar.br; 5 Graduate Program in Pharmaceutical Sciences, State University of Ponta Grossa, Ponta Grossa 84010-330, PR, Brazil; janemanfron@hotmail.com

**Keywords:** apigenin, flavone, mesenteric vascular bed, potassium channel, vasodilation

## Abstract

Background: Apigenin (4′,5,7-trihydroxyflavone), a flavonoid with potential cardiovascular benefits, has unclear mechanisms of action. This study investigates its effects on vascular function in Spontaneously Hypertensive Rats (SHRs). Methods: Mesenteric vascular beds (MVBs) were isolated from SHRs and perfused with increasing doses of apigenin after pre-contraction with phenylephrine. To explore the mechanisms, different MVBs were pre-perfused with antagonists and inhibitors, including indomethacin, L-NAME, and potassium channel blockers (tetraethylammonium, a non-specific potassium channel blocker; glibenclamide, an ATP-sensitive potassium channel blocker; 4-aminopyridine, a voltage-gated potassium channel blocker; charybdotoxin a selective intermediate-conductance calcium-activated potassium channel blocker; and apamin, a selective small-conductance calcium-activated potassium channel blocker). Results: Apigenin induced a dose-dependent reduction in perfusion pressure in MVBs with intact endothelium, an effect abolished by endothelium removal. L-NAME reduced apigenin-induced vasodilation by approximately 40%. The vasodilatory effect was blocked by potassium chloride and tetraethylammonium. The inhibition of small and intermediate calcium-activated potassium channels with charybdotoxin and apamin reduced apigenin-induced vasodilation by 50%, and a combination of these blockers with L-NAME completely inhibited the effect. Conclusions: Apigenin promotes vasodilation in resistance arteries through endothelial nitric oxide and calcium-activated potassium channels. These findings suggest that apigenin could have therapeutic potential in cardiovascular disease, warranting further clinical research.

## 1. Introduction

**Figure 1:**
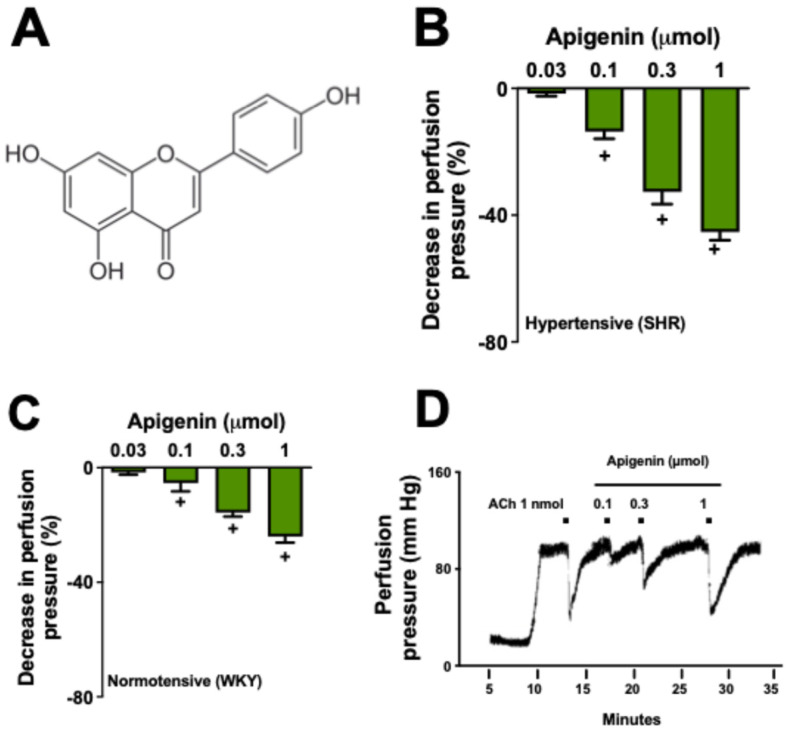
Apigenin induces vasodilation in resistance arteries of rats. Molecular structure of apigenin (**A**). Effects of apigenin on the mesenteric vascular beds of WKY and SHRs (**B**,**C**). Typical representative record of the administration of acetylcholine (1 nmol) and apigenin (0.1, 0.3, and 1 µmol) in preparations of mesenteric vascular bed of SHRs (**D**). Values represent the mean ± standard error of the mean (*n* = 6 preparations). + *p* < 0.05 compared with the previously administered dose. All experiments were performed in endothelium-intact preparations.

Apigenin (4′,5,7-trihydroxyflavone; Figure 1A) belongs to the flavone subclass and is the aglycone of several glycosides [1]. It is distributed in the plant kingdom, mainly in the species of the Asteraceae family such as *Artemisia* [2], *Baccharis* [3], and *Matricaria* [4]. In past years, many flavonoids, such as apigenin, have been identified as possible new medicines for cardiovascular diseases [5,6,7]. Apigenin has garnered significant commercial interest, primarily due to its use as a dietary supplement [8].

Apigenin has been associated with a range of biological effects, particularly due to its role as an antioxidant. It acts as a scavenger of reactive oxygen species (ROSs), reactive nitrogen species (RNSs), and phenoxyl radicals [9]. Studies have also shown that apigenin can reduce H_2_O_2_-induced DNA changes in lymphocytes [10], decrease cellular ROS levels, and inhibit both glutathione depletion and H_2_O_2_-induced lipid oxidation [11].

While the antioxidant properties of several flavonoids are often linked to in vitro studies, the antioxidant effects of apigenin have been demonstrated through in vivo methods as well [12]. Importantly, both in vivo and in vitro studies have shown that apigenin possesses biological properties beyond the regulation of cellular redox balance. These include anti-inflammatory (antiphlogistic) [13,14] and antineoplastic effects [15,16]. Previous reports indicate that the systemic administration of apigenin reduces blood pressure and attenuates cardiac hypertrophy and fibrosis in spontaneously hypertensive rats [17]. Additionally, cardioprotective effects have been observed following apigenin administration in experimental models of renal hypertension [18].

Although apigenin has shown potential cardiorenal effects, the molecular targets involved in these activities remain unidentified. In this study, spontaneously hypertensive rats (SHRs) were used to test the hypothesis that apigenin induces direct vasodilation in resistance arteries, which play a key role in blood pressure regulation. Additionally, we investigated the molecular mechanisms underlying apigenin’s vasodilatory effect.

## 2. Results

### 2.1. Vasodilatory Response of Apigenin in Resistance Arteries

To ensure the stability of phenylephrine-induced vascular tone throughout the experiments, we monitored the baseline tone and observed no significant reductions during apigenin administration. Prior to the administration of any substance, the perfusion of the preparations was 28 ± 4.2 mm Hg. The perfusion of phenylephrine (3 µM) elevated the pressure in the preparations to 108 ± 8.4 mm Hg, remaining stable for four hours. The administration of various doses of apigenin in mesenteric vascular beds (MVBs), pre-contracted with phenylephrine (3 µM), reduced perfusion pressure in a dose-dependent manner (Figure 1B,C). It was possible to demonstrate that the vasodilation obtained in the resistance arteries of hypertensive rats was significantly higher than that found in normotensive animals. Thus, for the continuation of our study, we chose to investigate the effects of apigenin only in arteries from SHRs.

### 2.2. Involvement of Nitric Oxide in the Vasodilatory Effects of Apigenin

**Figure 2:**
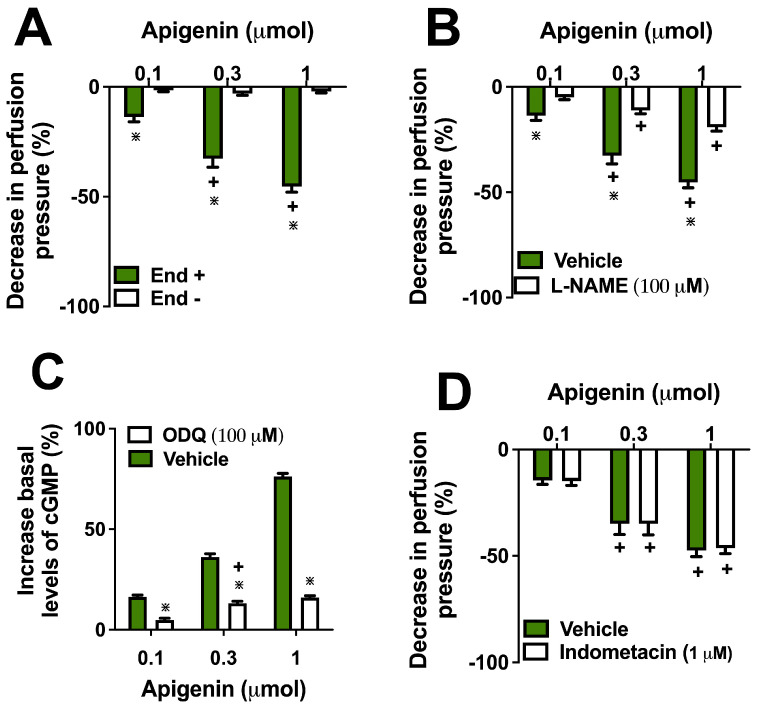
The vasodilatory effects of apigenin are dependent on vascular endothelium and nitric oxide release. Vasodilatory action of apigenin in the presence (End+) and absence of endothelium (End-) are presented (**A**). Apigenin’s vasodilatory action was investigated in the presence of the nitric oxide synthase inhibitor (L-NAME) (**B**), regarding the formation of cGMP (**C**), or during the inhibition of the enzyme cyclooxygenase (indomethacin) (**D**). Values represent the mean ± standard error of the mean (*n* = 6 preparations). * *p* < 0.05 compared with preparations in the presence of endothelium (**A**) or after treatment only with vehicle (**B**,**C**). + *p* < 0.05 compared with the respective previous dose.

To examine the role of nitric oxide (NO) in apigenin-induced vasodilation, we used L-NAME, a well-established nitric oxide synthase (NOS) inhibitor. By inhibiting NOS, we aimed to determine the extent to which NO mediates the observed vasodilatory effect of apigenin in mesenteric resistance arteries. Additionally, we also measured the tissue concentration of cGMP after sustained perfusion with apigenin. Finally, indomethacin was included to block cyclooxygenase (COX) activity, thereby allowing us to isolate the contributions of NO by eliminating the potential influence of COX-derived prostanoids. Pre-perfusion with deoxycholic acid, a chemical agent used to remove the endothelium, reduced the vasodilatory response to apigenin (0.1, 0.3, and 1 µmol) by 99% (Figure 2A). Conversely, L-NAME perfusion reduced the vasodilatory effects of all apigenin doses by approximately 40% (Figure 2B). As expected, the NO-donor SNP increased cGMP levels by approximately 202%, whereas co-incubation with ODQ (100 µM) completely prevented the SNP-mediated increases in cGMP. Incubation with apigenin (0.1, 0.3, and 1 µmol) increased cGMP levels by approximately 17%, 38%, and 76%, respectively, when compared with basal levels, whereas its co-incubation with ODQ completely abolished this effect (Figure 2C). Pre-perfusion with indomethacin did not affect the vascular response to apigenin (Figure 2D).

### 2.3. Involvement of K^+^ Channels in the Vasodilatory Effects of Apigenin

**Figure 3:**
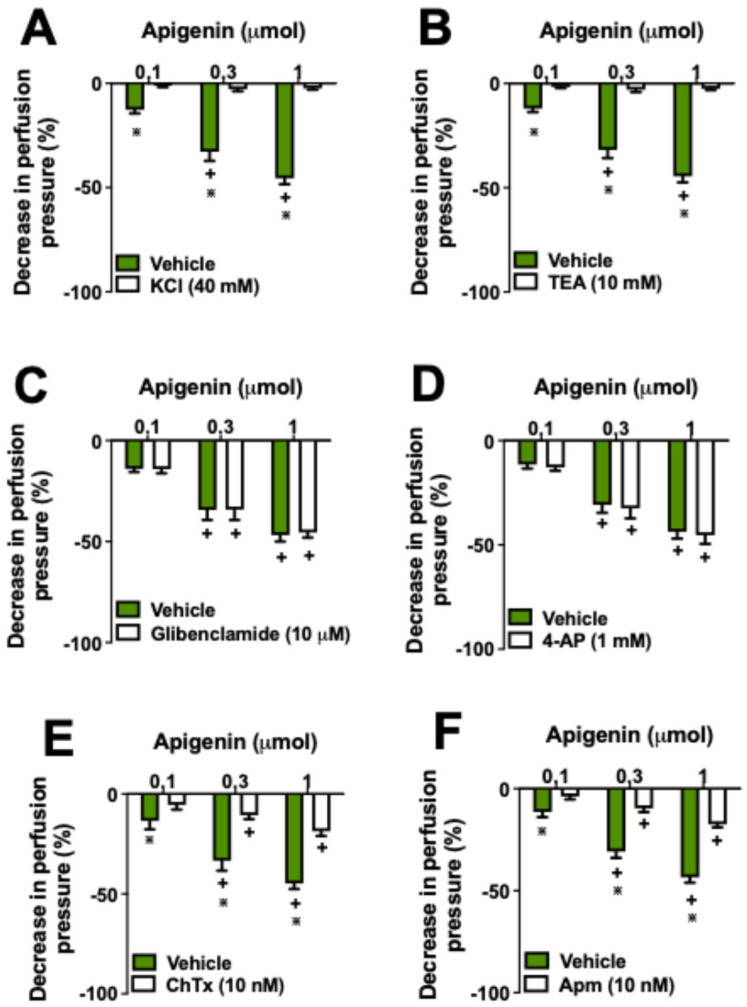
The vasodilatory effects of apigenin depend on potassium channels. Apigenin’s vasodilatory action was investigated in the presence of 40 mM KCl (**A**), tetraethylammonium (TEA) (**B**), glibenclamide (**C**), 4-aminopyridine (4-AP) (**D**), charybdotoxin (ChTx) (**E**), or apamin (Apm) (**F**). The results show the mean ± S.E.M. of six preparations per group. * Indicate *p* < 0.05 compared with the effects of apigenin on the vehicle group. + indicates *p* < 0.05 compared with the respective previous dose. All experiments were performed in endothelium-intact preparations.

Our initial results indicated that NO release plays a significant role in apigenin-induced vasodilation, leading us to investigate the involvement of calcium-activated potassium (KCa) channels, as these channels are known to mediate vasodilatory responses influenced by endothelial-derived factors. By assessing the effects of apigenin in the presence of potassium channel inhibitors, we sought to explore if these channels contribute to the vasorelaxation observed. The vasodilatory effects of apigenin were completely inhibited in the presence of 40 mM KCl or following pre-perfusion with tetraethylammonium (TEA), a nonselective blocker of KCa channels (Figure 3A,B). Pre-treatment with glibenclamide (an ATP-sensitive potassium channel blocker) or 4-aminopyridine (a voltage-gated potassium channel blocker) did not affect apigenin-induced vasodilation (Figure 3C,D). Blockers of IK (intermediate-conductance) and SK (small-conductance) KCa channels, charybdotoxin (ChTx) and apamin (Apm), respectively, reduced vasodilation induced by all doses of apigenin by 50% (Figure 3E,F).

**Figure 4:**
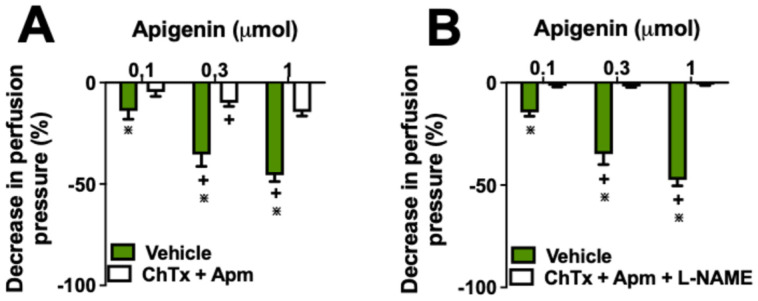
The vasodilator effects of apigenin are dependent on the release of nitric oxide and the activation of potassium channels. Apigenin’s vasodilatory action was investigated in the presence of charybdotoxin (ChTx) plus apamin (Apm) (**A**), and charybdotoxin (ChTx) plus apamin (Apm) plus L-NAME (**B**). The results show the mean ± S.E.M. of six preparations per group. * Indicate *p* < 0.05 compared with the effects of apigenin on the vehicle group. + indicates *p* < 0.05 compared with the respective previous dose. All experiments were performed in endothelium-intact preparations.

To further elucidate the interplay between NO and KCa channels, we combined L-NAME with KCa channel inhibitors, ChTx and Apm. The aim of these combinations was to clarify the respective contributions of NO and potassium channel activation to the vasodilatory effects of apigenin. Notably, ChTx + Apm did not produce an additive effect, suggesting that both KCa channels and NO independently mediate components of the vasodilation. To evaluate this hypothesis, we explored the inhibition effects of combined ChTx+L-NAME and Apm+L-NAME treatments. Interestingly, simultaneous treatment with ChTx, Apm, and L-NAME completely abolished vasorelaxation induced by all doses of apigenin (Figure 4B), whereas the combination of ChTx and Apm alone reduced apigenin’s effects by approximately 60% (Figure 4A).

## 3. Discussion

In this study, apigenin, a phenolic compound previously described in a wide range of medicinal and edible plants [1,2,3,4], was shown to reduce the tone of resistance arteries, which play a crucial role in regulating blood pressure [19]. Flavonoids, such as apigenin, trigger the release of nitric oxide (NO) and other endothelial mediators by activating endothelial nitric oxide synthase (eNOS). This process is initiated by an increase in intracellular calcium, which activates eNOS through the calcium/calmodulin pathway. Additionally, eNOS can be phosphorylated by kinases such as Akt. Furthermore, apigenin may enhance the production of prostacyclin and endothelium-derived hyperpolarizing factors, collectively promoting vasodilation through various signaling pathways [20,21,22]. These findings suggest that apigenin may act as a direct vasodilator, contributing to the cardiovascular effects already attributed to this compound and to medicinal species rich in this flavonoid [23,24].

In recent decades, there has been an increasing focus on the role of endothelium-derived vasoactive mediators in the cardiovascular field [25]. The endothelium regulates vascular homeostasis by synthesizing and releasing substances that either constrict or relax blood vessels in response to various stimuli, including chemical (both internal and external) and physical factors, such as shear stress and pulsatile stretch [26]. Endothelium-dependent mediators that activate potassium channels in vascular smooth muscle cells include nitric oxide (NO), prostacyclin, and endothelium-derived hyperpolarizing factors. These mediators facilitate muscle relaxation by activating small-conductance and intermediate-conductance calcium-activated potassium channels [27].

Nitric oxide and prostacyclin produced by endothelial cells significantly influence the tone of large arteries, whereas endothelium-derived hyperpolarizing factors (EDRFs) are crucial for regulating smaller resistance arteries, such as those found in the MVBs [28]. Our study demonstrated that damage to the endothelium inhibited the effects of all doses of apigenin, indicating a direct role of the endothelium in the vasodilator response. Notably, the endothelium-dependent effects observed in our experiments were not influenced by the inhibition of cyclooxygenase with indomethacin [28], but were partially reduced by the nitric oxide synthase inhibitor L-NAME [29]. These findings suggest that apigenin induces vascular relaxation in resistance arteries through endothelium-dependent mechanisms that are partially modulated by the release of nitric oxide. If we consider the absence of the effects of indomethacin on the response to apigenin, it is possible to hypothesize a residual participation of EDRFs in the vasodilatory response of this flavonoid.

To investigate the downstream pathways involved in the endothelium-dependent activity of apigenin, we conducted experiments using a potassium-mediated depolarization condition (high KCl; 40 mM). This condition resulted in the suppression of potassium currents across cellular membranes [30]. The potential blocking of these potassium currents prevented the decrease in apigenin-induced vasodilation, suggesting that the regulation of potassium efflux plays a significant role in the vascular response. To test this hypothesis, we utilized tetraethylammonium, a potassium channel blocker [31], which completely reversed the vascular effects of all doses of apigenin. Additionally, while not as effective as tetraethylammonium, selective blockers of intermediate and small-conductance calcium-activated potassium channels—charybdotoxin and apamin, respectively—also reduced the effects of apigenin [32]. The influence of glibenclamide and apamin was primarily observed in potassium channel modulation, suggesting that apigenin may interact with multiple signaling pathways involving these channels. This interaction could explain the partial modulation observed in the vasodilatory response to apigenin. Our data indicate that both types of calcium-dependent potassium channels may contribute to the vasodilatory response induced by apigenin. The molecular effects of NO may involve an increase in intracellular calcium levels, which subsequently activates eNOS via the calcium/calmodulin pathway [21], as well as the direct activation of potassium channels [33]. Given that there is no response to apigenin after treatment with charybdotoxin combined with apamin and L-NAME, it is reasonable to suggest that nitric oxide, as well as intermediate and small-conductance calcium-activated potassium channels, play a role in the endothelium-dependent effects of apigenin in the resistance arteries of SHRs. Therefore, all these results suggest that, among several EDRFs, the NO/cGMP pathway may become the major vasodilation mechanism in small resistance arteries under hypertensive conditions [34]. Our research on the vasodilatory effects of apigenin is consistent with the findings reported by Ko et al. [35], who investigated the mechanisms of apigenin-induced vasodilation in the rat thoracic aorta. Furthermore, the inhibitory effects observed in our study align with the results of Je et al. [36], who examined the impact of apigenin on vascular contractility through calcium desensitization-related pathways. These comparisons highlight the effects of apigenin across various vascular models and provide additional support for our findings.

Our study highlights the vasodilatory actions of apigenin in a controlled ex vivo environment, providing a foundational understanding that could guide further research in more complex models and eventually support clinical applications. The simplicity of our model isolates specific effects of apigenin, which are often challenging to discern in vivo, underscoring the unique contribution of our findings within vascular pharmacology.

Previous studies, such as Gao et al. [17], have shown that apigenin can modulate NADPH oxidase-dependent pathways and reduce reactive oxygen species (ROSs) in hypertensive models. These complementary mechanisms may enhance the vasodilatory activity observed here, suggesting multiple therapeutic avenues for apigenin in cardiovascular treatment. Finally, our study provides crucial insights into the vasodilatory actions of apigenin within a controlled ex vivo environment. These findings contribute to a foundational understanding that could inform future research in more complex models and eventually lead to clinical applications. The simplicity of our model allows for the isolation of specific effects, which can be challenging to discern within in vivo systems, thus underscoring the unique value of our research in the broader context of vascular pharmacology.

## 4. Materials and Methods

### 4.1. Drugs

The following drugs, salts, and solutions were used: apigenin, phenylephrine, acetylcholine chloride, glibenclamide, indomethacin, tetraethylammonium bromide, Nω-nitro-L-arginine methyl ester (L-NAME), apamin, charybdotoxin, iberiotoxin, ODQ, sodium nitroprusside, sodium deoxycholate, NaHCO_3_, NaCl, MgSO_4_, KCl, KH_2_PO_4_, CaCl_2_, ethylenediaminetetraacetic acid (EDTA), dextrose (Sigma-Aldrich, Saint Louis, MO, USA), xylazine and ketamine hydrochloride (Syntec, São Paulo, SP, Brazil), and heparin (Hipolabor Pharmaceutical, Belo Horizonte, MG, Brazil).

### 4.2. Animals

Fourteen-week-old male Wistar Kyoto (WKY) and spontaneously hypertensive rats (SHRs; 300–320 g) were obtained from the central animal facility at the Federal University of Grande Dourados (UFGD, Brazil). The animals had free access to food and water under controlled conditions, which included a temperature of 22 ± 2 °C, humidity of 50 ± 10%, and a 12 h light/dark cycle (lights on at 07:00 AM). All protocols conducted in this study were approved by the ethics committee for the use of animals at UFGD (authorization 07/2020).

### 4.3. Removal and Preparation of Mesenteric Vascular Beds (MVBs)

Initially, the WKY and SHRs were anesthetized (ketamine and xylazine; 100 and 20 mg/kg, respectively; i.p.), and the MVBs were isolated and prepared for perfusion [37]. The preparations were placed in a water-jacketed organ bath at a temperature of 37 °C and aerated with a mixture of 95% O_2_ and 5% CO_2_, and perfused with physiological salt solution (PSS containing [mM]: NaCl 119; KCl 4.7; CaCl_2_ 2.4; MgSO_4_ 1.2; NaHCO_3_ 25.0; KH_2_PO_4_ 1.2; dextrose 11.1; and EDTA 0.03) at a flow rate of 4 mL/min. The viability of the preparations was confirmed by administering phenylephrine (30 nmol). Changes in the perfusion pressure (mm Hg) were measured by a pressure transducer connected to an acquisition system (PowerLab^®^) and its application program (Chart, v 7.1; both from ADI Instruments, Castle Hill, Australia).

### 4.4. Evaluation of the Effects of Apigenin on the MVBs

Preparations with functional endothelium were continuously perfused with physiological salt solution (PSS) containing phenylephrine (3 µM). After stabilizing the increased perfusion pressure, bolus injections of apigenin were administered at doses of 0.03, 0.1, 0.3, and 1 µmol. The subsequent dose was given only after the perfusion pressure returned to baseline levels (pre-treatment).

### 4.5. Evaluation of the Molecular Pathways Involved in the Vasodilatory Response Induced by Apigenin

To remove the vascular endothelium, some preparations were perfused with PSS containing sodium deoxycholate (1.8 mg/mL) for 30 s, as previously described [38]. After the infusion of sodium deoxycholate, the system was perfused with regular PSS for an additional 40 min to stabilize the preparations. The effectiveness of sodium deoxycholate in promoting endothelium removal was verified by the absence of a reduction in perfusion pressure following a bolus injection of acetylcholine (ACh, 1 nmol). Subsequently, a dose-response curve for apigenin (0.1, 0.3, and 1 µmol) was performed.

In another set of experiments, preparations with intact endothelium were perfused with physiological saline solution (PSS) containing 3 µM phenylephrine, along with various agents given alone or in combination as follows: L-NAME (100 µM; a non-selective nitric oxide synthase inhibitor), indomethacin (1 µM; a non-selective inhibitor of cyclooxygenases), KCl (40 mM), tetraethylammonium (10 mM; a non-specific potassium channel blocker), glibenclamide (10 µM; an ATP-sensitive potassium channel blocker), 4-aminopyridine (100 µM; a voltage-gated potassium channel blocker), charybdotoxin (10 nM; a selective intermediate-conductance calcium-activated potassium channel blocker), and apamin (10 nM; a selective small-conductance calcium-activated potassium channel blocker). After 15 min of perfusion with one of the aforementioned solutions, apigenin (0.1, 0.3, and 1 µmol) or the vehicle was administered.

To confirm the role of the NO–cGMP pathway in the vasodilator effects of apigenin, we measured the intracellular concentration of cyclic guanosine monophosphate (cGMP) using methods described by Estancial et al. [39]. For this, MVBs (*n* = 6) from SHRs were prepared for perfusion as described above. After 30 min of perfusion for stabilization, the MVBs were then stimulated for 15 min with sodium nitroprusside (SNP, an NO donor, 10 µm) or apigenin (0.1, 0.3, and 1 µmol) in the absence and presence of the soluble guanylyl cyclase (sGC) inhibitor ODQ (100 µm, 30 min). Subsequently, the tissues were removed and frozen in liquid nitrogen. The tissues were homogenized in trichloroacetic acid (5% wt/vol) and centrifuged (10 min at 4 °C at 1500× *g*), and the supernatant was collected. The pellet was dried and weighed, and the trichloroacetic acid was extracted. Procedures for antibody incubation and tracer preparation were performed as described in commercially available kits (Cayman Chemical Cyclic GMP EIA kit, Ann Arbor, MI, USA). All experiments were performed in duplicate.

### 4.6. Statistical Analysis

The data are presented as the mean and standard error of the mean for six preparations in each group. Statistical analysis was performed using analysis of variance (ANOVA) followed by the Bonferroni test. The established significance level was set at *p* ≤ 0.05. All analyses were conducted using GraphPad Prism 10 software for macOS (GraphPad Software, Boston, MA, USA).

## 5. Conclusions

This study demonstrated that the flavonoid apigenin, commonly found in various medicinal and edible plants, can induce endothelium-dependent vasodilatory effects in spontaneously hypertensive rats (SHRs). The vasodilation is mediated by nitric oxide and small and intermediate calcium-activated potassium channels. Further research is needed to assess the practical implications of these findings for the treatment of cardiovascular diseases. Such investigations could enhance our understanding of the potential benefits of using apigenin as an herbal remedy or dietary supplement.

## Data Availability

The data presented in this study are available on request from the corresponding author.
